# Artificial Intelligence as an ally to the Nursing Process: pathways and ethical considerations

**DOI:** 10.1590/1518-8345.0000.4867

**Published:** 2026-02-02

**Authors:** Isabelle Cristinne Pinto Costa, Elielza Guerreiro Menezes, Rodrigo Jensen, Viviane Martins da Silva

**Affiliations:** 1 Universidade Federal de Alfenas, Escola de Enfermagem, Alfenas, MG, Brazil.; 2 Universidade do Estado do Amazonas, Escola Superior de Ciências da Saúde, Manaus, AM, Brazil.; 3 Universidade de São Paulo, Escola de Enfermagem, São Paulo, SP, Brazil.; 4 Universidade Federal do Ceará, Faculdade de Farmácia, Odontologia e Enfermagem, Fortaleza, CE, Brazil.

**Figure:**
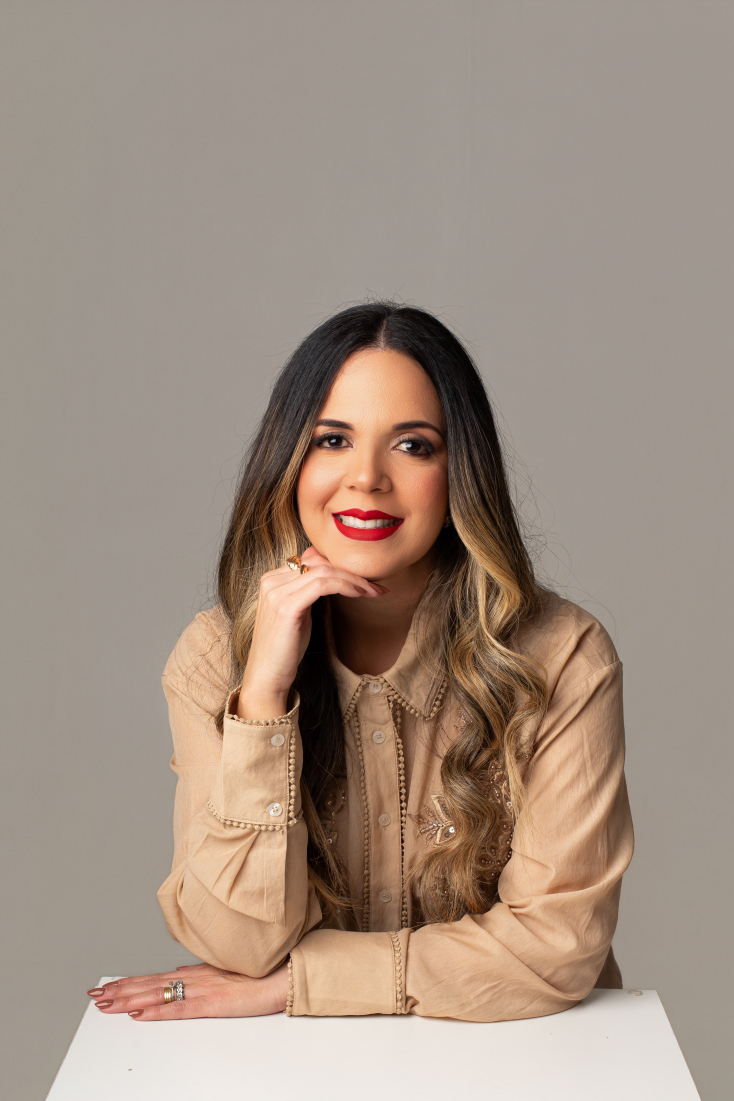

**Figure:**
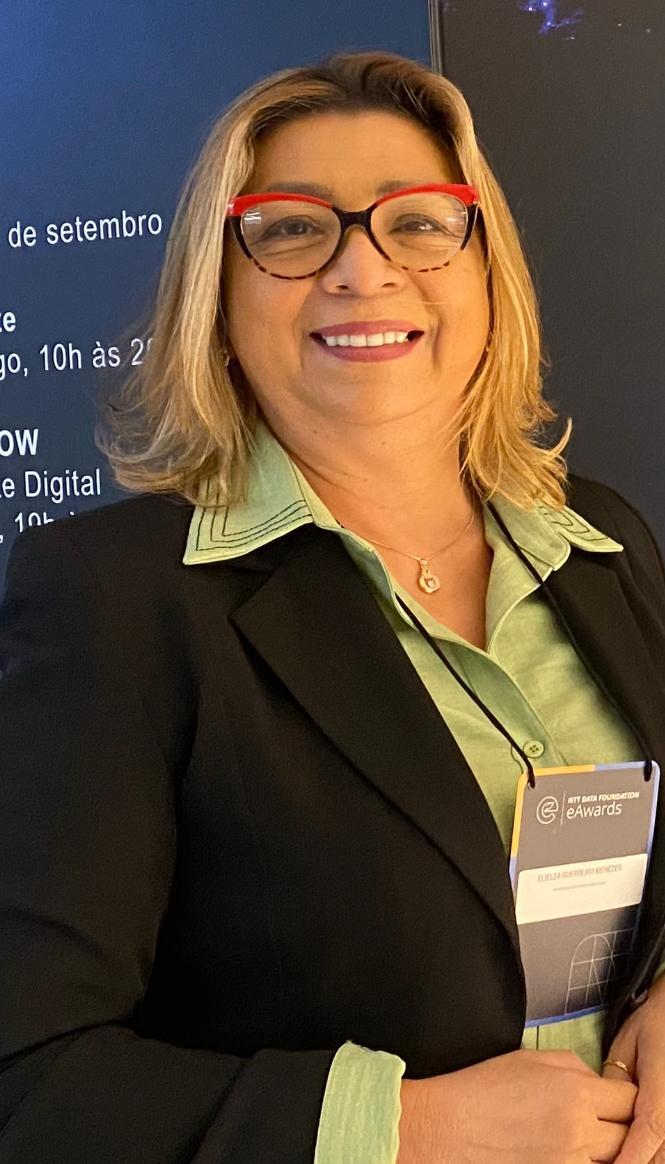

**Figure:**
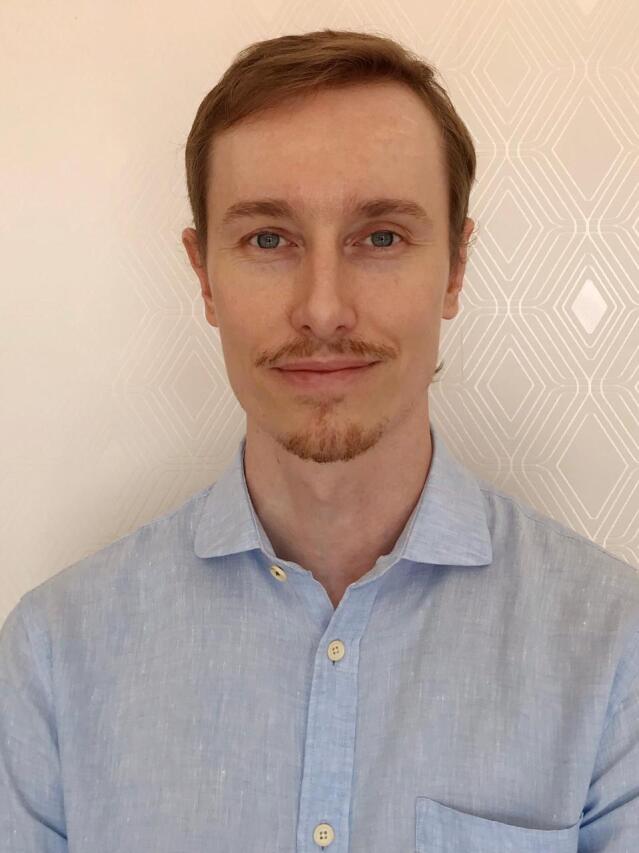

**Figure:**
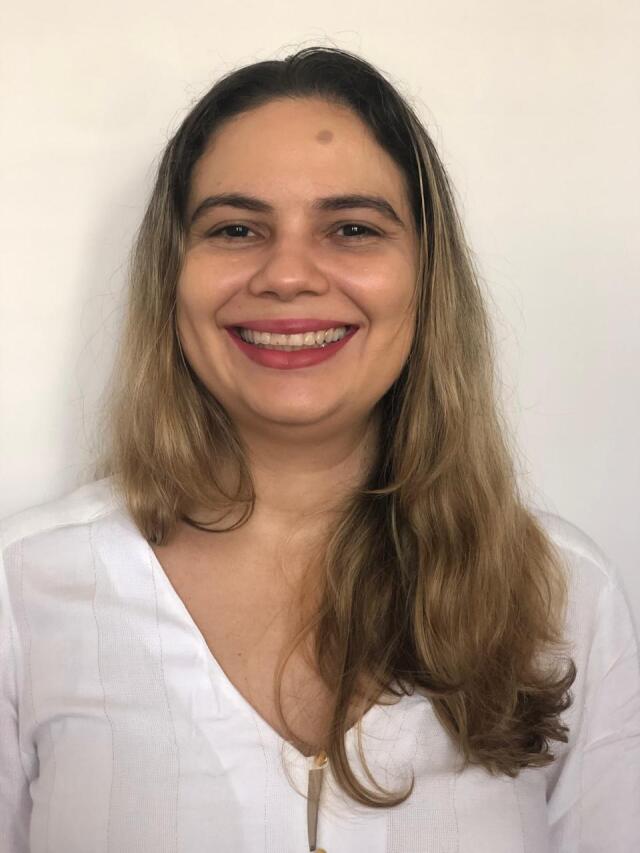

Health professionals are experiencing a new era in which lines of computational code intertwine with clinical decision-making. At the center of this convergence, Nursing — historically rooted in human contact — assumes a new role: leading digital workflows that enhance and expand patient care. The arrival of Artificial Intelligence (AI) reshapes the foundations of practice, offering unprecedented precision, agility, and personalization, while imposing the ethical imperative to reflect on its impacts, risks, and possibilities.

This debate is urgent given the pace of digital transformation in health and the Brazilian Federal Nursing Council (COFEN) Resolution No. 736/2024^(1)^, which updates the guidelines of the Nursing Process (NP) and recognizes the value of standardized terminologies and emerging technologies. At the same time, global organizations such as the World Health Organization (WHO)^(2)^ and the International Council of Nurses (ICN)^(3)^ reinforce normative frameworks geared toward the ethical, equitable, and safe adoption of AI. The invitation is clear: Brazilian Nursing must take the lead in this transition without abandoning the humanitarian essence that sustains its identity.

In the digital era, nurses encounter new possibilities for executing the steps of the NP. In the Nursing assessment phase, AI algorithms process, in real time, thousands of physiological parameters, detecting changes imperceptible to the human eye. Wearable sensors and Internet of Things (IoT) devices continuously capture data, while Natural Language Processing (NLP) transforms unstructured records into clinical indicators. In the Nursing Diagnosis phase, intelligent tools link data to terminologies such as NANDA International, Inc. (NANDA-I) and the International Classification for Nursing Practice (ICNP®), recommending diagnostic hypotheses and expanding the nurse’s clinical reasoning.

Nursing Planning is strengthened by predictive models that integrate scientific evidence, individual characteristics, and patient preferences, generating interventions based on the Nursing Interventions Classification (NIC) and goals grounded in the Nursing Outcomes Classification (NOC). Nursing Implementation may gain automation through assistive robots, devices, and mobile applications for bedside documentation. Nursing Evaluation may be supported by analytical dashboards that flag subtle clinical changes, consolidating a data-driven learning cycle.

Intelligent triage systems can anticipate clinical deterioration, detecting sepsis hours before conventional protocols. Voice recognition enables immediate documentation, freeing more time for direct care. Smart alerts reduce medication errors, and predictive workload models optimize human resources, contributing to the reduction of occupational stress. As a driver of critical thinking and clinical reasoning, AI brings together NLP and machine learning algorithms to predict risks of falls, pressure injuries, and readmissions, processing a volume of variables that exceed conventional human analysis. Integration with terminologies such as NANDA-I, NIC, NOC, and ICNP enhances suggested diagnoses, aligned interventions, and measurable results within seconds. International think tanks highlight that multiprofessional collaboration is a key condition for success in this journey^(4)^.

To fully benefit from these advances, Nursing professionals need new competencies. The nurse profile now includes data management skills, interpretation of AI-generated results, and collaborative work with automated systems. Digital literacy — previously limited to software use — now requires critical analysis of dashboards, interpretation of metrics such as sensitivity and overfitting, and the ability to question algorithmic outcomes. Ethics becomes central: biased data can generate discriminatory decisions, and sensitive information demands rigorous protection. The ICN^(3)^ highlights that nurses continue to advocate for patients’ rights in hypertechnological ecosystems. Investment in training programs is urgent, from undergraduate education to specialized programs in AI applied to health.

Despite its transformative potential, implementing AI in health faces technical challenges that may compromise its reliability. Among them, overfitting stands out as one of the most critical limitations of predictive machine-learning models. This phenomenon occurs when a model fits excessively to the training data, capturing noise and idiosyncratic characteristics. The result is artificially elevated performance in controlled environments, but ineffective outcomes in real-world settings, with direct impacts on patient safety. Thus, nurses must develop skills to critically analyze algorithmic responses and determine their validity in clinical practice.

The Brazilian General Data Protection Law (LGPD)^(5)^ requires anonymization, informed consent, and algorithmic transparency. Algorithmic bias may widen inequities: non-representative datasets impair performance for vulnerable populations. The WHO^(2)^ proposes six principles for ethical AI — autonomy, well-being, transparency, accountability, equity, and sustainability — principles echoed in COFEN Resolution 736/2024^(1)^, which reaffirms that nurses remain legally responsible for decision-making, even when using automated recommendations. Technology must therefore enhance, not replace, the nurse–patient relationship.

To realize the transformative potential of AI in the NP, three integrated fronts are proposed. Governance: creation of multidisciplinary committees to assess and monitor the performance, safety, and impact of implemented technologies, with active participation of Nursing professionals; as well as involving nurses in decision-making spaces on AI in health and in algorithm development, implementation, and evaluation. Translational research: promotion of multicenter studies to develop solutions sensitive to the Brazilian context in partnerships among universities, health services, and industry. Training: education on the ethical and responsible use of AI and data science at all levels of instruction, alongside specialized programs that ensure technical, reflective, and critical competence in the use of AI in health.

Brazilian Nursing faces a historic opportunity. If it succeeds in articulating technological innovation, ethics, and a commitment to care, the profession will not only accompany but may lead a new frontier in health care. Lines of code, allied to clinical competence, can enhance precision and expand the reach of nursing outcomes. This is the path for AI to become an ally of the NP and, above all, of the patients who trust in the science and sensitivity of those who care.

## References

[1] Conselho Federal de Enfermagem (BR) (2024). Resolução COFEN nº 736, de 17 de janeiro de 2024. Dispõe sobre a implementação do Processo de Enfermagem em todo contexto socioambiental onde ocorre o cuidado de enfermagem.

[2] World Health Organization (2021). Ethics and governance of artificial intelligence for health.

[3] International Council of Nurses (2023). Digital health transformation and nursing practice: Position Statement.

[4] Rony M. K. K., Parvin M. R., Ferdousi S. (2024). Advancing nursing practice with artificial intelligence: Enhancing preparedness for the future. Nurs Open.

[5] Brasil (2018). Lei nº 13.709, de 14 de agosto de 2018. Dispõe sobre a proteção de dados pessoais e altera a Lei nº 12.965, de 23 de abril de 2014.

