# Supplementary material for: Artificial Intelligence as an ally to the Nursing Process: pathways and ethical considerations
Source: Rev Lat Am Enfermagem. 2026 Feb 2;34:e4867. doi: 10.1590/1518-8345.0000.4867 (PMC12866941; doi:10.1590/1518-8345.0000.4867)
Supplement: Translation

# Translation: Inteligência artificial como aliada ao Processo de Enfermagem: caminhos e considerações éticas

**DOI:** 10.1590/1518-8345.0000.4868

**Authors:** Isabelle Cristinne Pinto Costa, Elielza Guerreiro Menezes, Rodrigo Jensen, Viviane Martins da Silva

**Affiliations:** 1 Universidade Federal de Alfenas, Escola de Enfermagem, Alfenas, MG, Brasil.; 2 Universidade do Estado do Amazonas, Escola Superior de Ciências da Saúde, Manaus, AM, Brasil.; 3 Universidade de São Paulo, Escola de Enfermagem, São Paulo, SP, Brasil.; 4 Universidade Federal do Ceará, Faculdade de Farmácia, Odontologia e Enfermagem, Fortaleza, CE, Brasil.

Profissionais da saúde vivenciam uma nova era, em que linhas de código computacional se entrelaçam com a tomada de decisão clínica. No centro dessa confluência, a Enfermagem — historicamente ligada ao contato humano — assume um novo papel: liderar fluxos digitais que qualificam e expandem o cuidado ao paciente. A chegada da Inteligência Artificial (IA) redesenha fundamentos da prática, oferecendo precisão, agilidade e personalização inéditas, mas impondo o imperativo ético de refletir sobre impactos, riscos e possibilidades.

Esse debate é urgente diante da velocidade da transformação digital em saúde e da Resolução COFEN nº 736/2024^(1)^, que atualiza diretrizes do Processo de Enfermagem (PE) e reconhece o valor das terminologias padronizadas e tecnologias emergentes. Em paralelo, organismos globais como a Organização Mundial da Saúde (OMS)^(2)^ e o *International Council of Nurses* (ICN)^(3)^ reforçam marcos normativos voltados à adoção ética, equitativa e segura da IA. O convite é claro: a Enfermagem brasileira deve assumir protagonismo nessa transição, sem abandonar a essência humanitária que sustenta sua identidade.

Na era digital, enfermeiros encontram novas possibilidades na execução das etapas do PE. Na etapa de avaliação de Enfermagem, algoritmos de IA processam, em tempo real, milhares de dados de parâmetros fisiológicos, detectando alterações imperceptíveis ao olhar humano. Sensores vestíveis e dispositivos de Internet das Coisas (IoT) capturam dados continuamente, enquanto o Processamento de Linguagem Natural (PLN) transforma registros não estruturados em indicadores clínicos. Na etapa de Diagnóstico de Enfermagem, ferramentas inteligentes relacionam dados com terminologias, como a NANDA *International, Inc.* (NANDA-I) e a Classificação Internacional para a Prática de Enfermagem (CIPE®), recomendando hipóteses diagnósticas e ampliando o espectro do raciocínio clínico do enfermeiro.

O Planejamento de Enfermagem é potencializado por modelos preditivos que combinam evidência científica, características individuais e preferências do paciente, gerando intervenções baseadas na Nursing Interventions Classification (NIC) e metas fundamentadas na Nursing Outcomes Classification (NOC). A Implementação de Enfermagem pode ganhar automação com robôs assistivos, dispositivos e aplicativos móveis para registro de intervenções à beira- leito. A Evolução de Enfermagem pode ser apoiada por *dashboards* analíticos, os quais sinalizam mínimas alterações clínicas, consolidando um ciclo de aprendizagem baseado em dados.

Sistemas de triagem inteligente podem antecipar a deterioração clínica, detectando sepse horas antes dos protocolos convencionais. O reconhecimento de voz permite registros imediatos, liberando mais tempo para o cuidado direto. Alertas inteligentes reduzem erros de medicação e modelos preditivos da carga de trabalho otimizam recursos humanos, contribuindo para a redução do estresse ocupacional.

Como motor do pensamento crítico e do raciocínio clínico, a IA alia o PLN aos algoritmos de aprendizado de máquina (*machine learning*) para prever risco de quedas, úlceras por pressão e reinternações, processando volume de variáveis que escapam à análise humana convencional. A integração com terminologias como NANDA-I, NIC, NOC e CIPE potencializa diagnósticos sugeridos, intervenções alinhadas e resultados mensuráveis em segundos. *Think tanks* internacionais reforçam que a colaboração multiprofissional é condição essencial para o sucesso dessa jornada^(4)^. Para usufruir plenamente desses avanços, os profissionais de Enfermagem precisam de novas competências.

O perfil do enfermeiro passa a incluir habilidades em gestão de dados, interpretação de resultados de IA e atuação colaborativa com sistemas automatizados. O letramento digital, antes limitado ao uso de *softwares*, agora inclui a análise crítica de *dashboards*, a interpretação de métricas como sensibilidade e sobre ajuste e a habilidade de questionar resultados algorítmicos. A ética torna-se central: dados enviesados podem gerar decisões preconceituosas; informações sensíveis demandam segurança rigorosa. O ICN^(3)^ destaca que o enfermeiro continua a advogar pelos direitos do paciente em ecossistemas hipertecnológicos. Urge investir em programas de formação e capacitação, desde a graduação até especializações em IA aplicada à saúde.

Apesar do potencial transformador, a implementação da IA na saúde enfrenta desafios técnicos que podem comprometer sua confiabilidade. Entre eles, o *overfitting* — ou sobreajuste — destaca-se como uma das limitações mais críticas dos modelos preditivos baseados em *machine learning*. Esse fenômeno ocorre quando um modelo se ajusta excessivamente aos dados de treinamento, capturando ruídos e características idiossincráticas específicas. O resultado é um desempenho artificialmente elevado em ambientes controlados, mas ineficaz em contextos reais, com impactos diretos na segurança do paciente. Diante disso, são necessárias habilidades para uma análise crítica das respostas geradas pelos modelos algoritmos, a determinar sua validade na prática clínica.

A Lei Geral de Proteção de Dados Pessoais (LGPD)^(5)^ exige anonimização, consentimento informado e transparência algorítmica. O viés algorítmico pode ampliar desigualdades: bases de dados não representativas prejudicam o desempenho para populações vulneráveis. A OMS^(2)^ propõe seis princípios para uma IA ética: autonomia, bem- estar, transparência, responsabilidade, equidade e sustentabilidade, princípios que encontram eco na Resolução COFEN 736/2024^(1)^, ao reafirmar que o enfermeiro responde legalmente pela tomada de decisão, mesmo diante de recomendações automatizadas. A tecnologia, portanto, deve potencializar, e não substituir a relação enfermeiro-paciente.

Para concretizar o potencial transformador da IA no PE, sugerem-se três frentes integradas. Governança: criação de comitês multidisciplinares que avaliem e monitorem o desempenho, a segurança e o impacto das tecnologias adotadas, com participação ativa de profissionais de Enfermagem; assim como o envolvimento dos enfermeiros nos espaços de decisórios sobre IA em saúde e na construção de modelos algoritmos, implementação e avaliação. Pesquisa translacional: fomento de estudos multicêntricos que desenvolvam soluções sensíveis ao contexto brasileiro, com parceria entre universidades, serviços de saúde e indústria. Capacitação: ensino sobre o uso ético e responsável da IA e de ciência de dados em todos os níveis formativos, além da oferta de especializações específicas, a garantir competência técnica, reflexiva e crítica sobre o uso da IA na saúde.

A Enfermagem brasileira tem diante de si uma oportunidade histórica. Se conseguir articular a inovação tecnológica, a ética e o compromisso com o cuidado, a profissão não apenas acompanhará, mas poderá liderar uma nova fronteira do cuidado em saúde. Linhas de código aliadas às competências clínicas podem ampliar a precisão no cuidado e expandir o alcance dos resultados de enfermagem. Esse é o caminho para que a IA se torne aliada do PE e, sobretudo, dos pacientes que confiam na ciência e na sensibilidade de quem cuida.

# Translation: La inteligencia artificial como aliada del Proceso de Enfermería: caminos y consideraciones éticas

**DOI:** 10.1590/1518-8345.0000.4866

**Authors:** Isabelle Cristinne Pinto Costa, Elielza Guerreiro Menezes, Rodrigo Jensen, Viviane Martins da Silva

**Affiliations:** 1 Universidade Federal de Alfenas, Escola de Enfermagem, Alfenas, MG, Brasil.; 2 Universidade do Estado do Amazonas, Escola Superior de Ciências da Saúde, Manaus, AM, Brasil.; 3 Universidade de São Paulo, Escola de Enfermagem, São Paulo, SP, Brasil.; 4 Universidade Federal do Ceará, Faculdade de Farmácia, Odontologia e Enfermagem, Fortaleza, CE, Brasil.

Los profesionales de la salud viven una nueva era, en la que líneas de código computacional se entrelazan con la toma de decisiones clínicas. En el centro de esta convergencia, la Enfermería - históricamente vinculada al contacto humano - asume un nuevo papel: liderar flujos digitales que cualifican y expanden el cuidado al paciente. La llegada de la Inteligencia Artificial (IA) redibuja los fundamentos de la práctica, ofreciendo una precisión, agilidad y personalización inéditas, pero imponiendo el imperativo ético de reflexionar sobre sus impactos, riesgos y posibilidades.

Este debate es urgente ante la velocidad de la transformación digital en salud y la Resolución COFEN nº 736/2024^(1)^, que actualiza las directrices del Proceso de Enfermería (PE) y reconoce el valor de las terminologías estandarizadas y de las tecnologías emergentes. Paralelamente, organismos internacionales como la Organización Mundial de la Salud (OMS)^(2)^ y el *International Council of Nurses* (ICN)^(3)^ refuerzan marcos normativos orientados a la adopción ética, equitativa y segura de la IA. El llamado es claro: la Enfermería brasileña debe asumir el protagonismo en esta transición sin abandonar la esencia humanitaria que sustenta su identidad.

En la era digital, los enfermeros encuentran nuevas posibilidades para ejecutar las etapas del PE. En la evaluación de Enfermería, algoritmos de IA procesan, en tiempo real, miles de datos fisiológicos, detectando alteraciones imperceptibles al ojo humano. Sensores portátiles y dispositivos de Internet de las Cosas (IoT) capturan datos continuamente, mientras el Procesamiento del Lenguaje Natural (PLN) transforma registros no estructurados en indicadores clínicos. En el Diagnóstico de Enfermería, herramientas inteligentes relacionan datos con terminologías como NANDA *International, Inc.* (NANDA-I) y la Clasificación Internacional para la Práctica de Enfermería (CIPE®), recomendando hipótesis diagnósticas y ampliando el espectro del razonamiento clínico del enfermero.

La Planificación de Enfermería se ve fortalecida por modelos predictivos que integran evidencia científica, características individuales y preferencias del paciente, generando intervenciones basadas en la *Nursing Interventions Classification* (NIC) y metas fundamentadas en la *Nursing Outcomes Classification* (NOC). La Implementación de Enfermería puede contar con automatización mediante robots asistivos, dispositivos y aplicaciones móviles para el registro de intervenciones al pie de la cama. La Evolución de Enfermería puede apoyarse en paneles analíticos que señalan mínimas alteraciones clínicas, consolidando un ciclo de aprendizaje basado en datos.

Los sistemas de triaje inteligente pueden anticipar la deterioración clínica, detectando sepsis horas antes de los protocolos convencionales. El reconocimiento de voz permite registros inmediatos, liberando más tiempo para el cuidado directo. Las alertas inteligentes reducen errores de medicación, y los modelos predictivos de carga laboral optimizan recursos humanos, contribuyendo a la reducción del estrés ocupacional.

Como motor del pensamiento crítico y del razonamiento clínico, la IA articula el PLN con algoritmos de aprendizaje automático (*machine learning*) para predecir riesgos de caídas, úlceras por presión y reingresos, procesando un volumen de variables que excede la capacidad del análisis humano convencional. La integración con terminologías como NANDA-I, NIC, NOC y CIPE potencia diagnósticos sugeridos, intervenciones alineadas y resultados mensurables en segundos. *Think tanks* internacionales refuerzan que la colaboración multiprofesional es condición esencial para el éxito de esta trayectoria^(4)^.

Para aprovechar plenamente estos avances, los profesionales de Enfermería necesitan nuevas competencias. El perfil del enfermero pasa a incluir habilidades en gestión de datos, interpretación de resultados de IA y actuación colaborativa con sistemas automatizados. El alfabetismo digital - antes limitado al uso de *software* - ahora incluye el análisis crítico de paneles, la interpretación de métricas como sensibilidad y sobreajuste, y la habilidad de cuestionar resultados algorítmicos. La ética se vuelve central: datos sesgados pueden generar decisiones discriminatorias; informaciones sensibles requieren seguridad rigurosa. El ICN^(3)^ destaca que el enfermero continúa abogando por los derechos del paciente en ecosistemas hipertecnológicos. Es urgente invertir en programas de formación y capacitación, desde el pregrado hasta especializaciones en IA aplicada a la salud.

A pesar de su potencial transformador, la implementación de la IA en salud enfrenta desafíos técnicos que pueden comprometer su confiabilidad. Entre ellos, el *overfitting* - o sobreajuste - se destaca como una de las limitaciones más críticas de los modelos predictivos basados en *machine learning*. Este fenómeno ocurre cuando el modelo se ajusta excesivamente a los datos de entrenamiento, capturando ruidos y características idiosincráticas. El resultado es un rendimiento artificialmente elevado en entornos controlados, pero ineficaz en contextos reales, con impactos directos en la seguridad del paciente. Ante esto, se requieren habilidades para el análisis crítico de las respuestas generadas por los modelos algorítmicos, determinando su validez en la práctica clínica.

La Ley General de Protección de Datos Personales (LGPD)^(5)^ exige anonimización, consentimiento informado y transparencia algorítmica. El sesgo algorítmico puede ampliar desigualdades: bases de datos no representativas perjudican el desempeño en poblaciones vulnerables. La OMS^(2)^ propone seis principios para una IA ética: autonomía, bienestar, transparencia, responsabilidad, equidad y sostenibilidad, principios que se reflejan en la Resolución COFEN 736/2024^(1)^, al reafirmar que el enfermero es legalmente responsable por la toma de decisiones, incluso ante recomendaciones automatizadas. La tecnología, por lo tanto, debe potenciar—y no sustituir—la relación enfermero- paciente.

Para materializar el potencial transformador de la IA en el PE, se proponen tres frentes integradas. Gobernanza: creación de comités multidisciplinarios que evalúen y monitoreen el desempeño, la seguridad y el impacto de las tecnologías adoptadas, con participación activa de profesionales de Enfermería; así como la inclusión de enfermeros en los espacios decisorios sobre IA en salud y en la construcción, implementación y evaluación de modelos algorítmicos. Investigación traslacional: fomento de estudios multicéntricos que desarrollen soluciones sensibles al contexto brasileño, en alianza entre universidades, servicios de salud y la industria. Capacitación: formación sobre el uso ético y responsable de la IA y la ciencia de datos en todos los niveles educativos, además de ofertas de especializaciones específicas que garanticen competencias técnicas, reflexivas y críticas sobre el uso de la IA en salud.

La Enfermería brasileña tiene ante sí una oportunidad histórica. Si logra articular innovación tecnológica, ética y compromiso con el cuidado, la profesión no solo acompañará, sino que podrá liderar una nueva frontera del cuidado en salud. Líneas de código, aliadas a competencias clínicas, pueden ampliar la precisión del cuidado y expandir el alcance de los resultados de enfermería. Este es el camino para que la IA se convierta en aliada del PE y, especialmente, de los pacientes que confían en la ciencia y en la sensibilidad de quienes cuidan.
